# The ADAM Family of Proteases: Structure, Substrates, and Roles in Liver Diseases

**DOI:** 10.3390/ijms27041626

**Published:** 2026-02-07

**Authors:** Yufei Chen, Runxuan Zhou, Tinghui Zhao, Dong Xiang, Xuepeng Gong

**Affiliations:** 1 Department of Pharmacy, Tongji Hospital, Tongji Medical College, Huazhong University of Science and Technology, Wuhan 430030, China; m202376494@hust.edu.cn; 2 Cancer Center, Tongji Hospital, Tongji Medical College, Huazhong University of Science and Technology, Wuhan 430030, China; m202376689@hust.edu.cn; 3 Department of Pharmacy, Wuhan Mental Health Center, Wuhan 430030, China; zhaothwzk@163.com

**Keywords:** ADAM proteases, liver diseases, ectodomain shedding, molecular mechanisms, therapeutic targeting

## Abstract

The ADAM (a disintegrin and metalloproteinase) family, a class of transmembrane proteases with multiple biological functions, plays pivotal roles in processes of proteolytic ectodomain shedding, which are enabled by its unique structural characteristics. In recent years, advancements in molecular biology techniques have led to the progressive identification of shed substrates from ADAM members, whose aberrant expression or dysregulation is closely implicated in the initiation and progression of liver diseases. This review systematically outlines the core domain architecture and biological functions of ADAM proteases, summarizes their major shedding substrates, and elaborates the molecular mechanisms by which the ADAM members regulate the pathophysiological processes of liver diseases. By synthesizing current research advances and unresolved challenges, this work aims to establish a theoretical foundation and propose future research directions for the development of ADAM-based diagnostic markers, targeted therapeutics, and clinical translation in liver diseases.

## 1. Introduction

The ADAM family represents a group of type I transmembrane glycoproteins that belong to the metzincin superfamily of zinc-dependent metalloproteinases. To date, more than 40 ADAM members have been identified in mammals, with approximately half of them possessing functional catalytic activity [1]. The nomenclature “A Disintegrin And Metalloproteinase” aptly describes their core structural domains: a prodomain, a metalloproteinase domain, a disintegrin domain, a cysteine-rich region, an epidermal growth factor (EGF)-like domain, a transmembrane domain, and a cytoplasmic tail [2]. This sophisticated multidomain architecture equips ADAM proteases with the capacity to mediate both proteolytic and adhesive interactions [3]. Their most characterized function is “ectodomain shedding,” a pivotal post-translational process whereby ADAMs cleave the extracellular regions of a diverse array of membrane-anchored proteins, including cytokines, growth factors, receptors, and adhesion molecules [4,5]. This shedding activity acts as a master switch, regulating the bioavailability and function of these substrates, thereby profoundly influencing crucial cellular processes such as signaling, adhesion, proliferation, and migration [3]. Consequently, ADAM proteases are fundamental to a wide spectrum of physiological events, including development, immunity, and tissue repair [6].

The liver, the largest internal organ, is essential for maintaining metabolic homeostasis through its critical roles in metabolism, synthesis, storage, secretion, and immunity. Liver diseases encompass a variety of disorders affecting the liver, such as viral hepatitis, alcoholic liver disease, non-alcoholic fatty liver disease, drug-induced liver injury, autoimmune liver diseases, hereditary liver diseases, liver cirrhosis, and liver cancer. The progression of these diseases typically begins with initial hepatocyte damage and inflammation, advances to collagen-deposited hepatic fibrosis, and may ultimately develop into liver cirrhosis or even hepatocellular carcinoma (HCC) if left untreated. Liver diseases result in over 2 million deaths globally each year, accounting for 4% of all deaths worldwide, and representing the 11th leading cause of mortality [7]. While current management strategies (e.g., pharmacological interventions and lifestyle modifications) offer benefits, they often have limitations in halting disease progression. Therefore, elucidating the molecular mechanisms underlying liver disease pathogenesis has become crucial. In this complex pathogenesis, post-translational modifications, particularly ectodomain shedding mediated by proteases like the ADAM family, have emerged as critical regulatory layers.

Recent studies increasingly reveal that the ADAM protease family plays a crucial role in the pathophysiological networks of liver diseases. ADAM protease-mediated extracellular domain shedding events are deeply involved in regulating hepatocyte injury and death, inflammation, fibrosis, and the development of HCC. However, the specific mechanisms of action, substrate profiles, and interrelationships of different ADAM family members in various liver diseases have not been systematically elucidated. Therefore, this review aims to systematically integrate current knowledge regarding the structure, substrates, and role of ADAM family members in liver diseases, and to discuss the translational potential of targeting ADAM proteases in future liver disease management.

## 2. Structural Features and Substrates of ADAM Proteases

### 2.1. Structure and Biological Functions of ADAM Proteins

**Figure 1:**
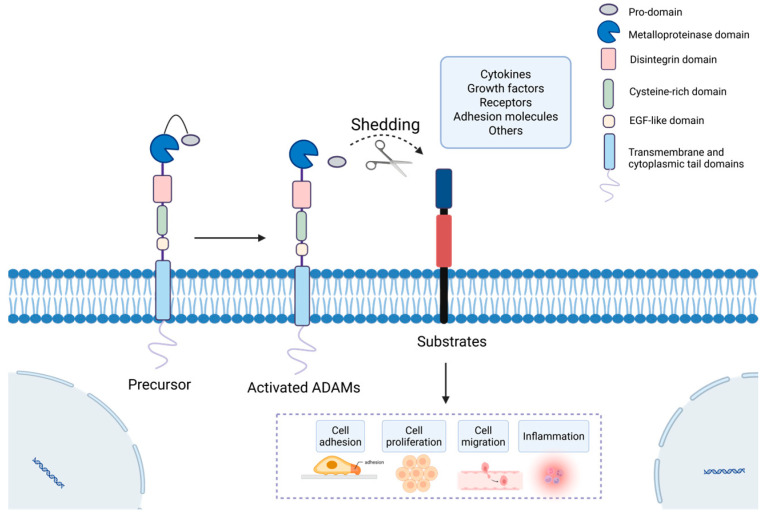
Domain structure and biological functions of ADAM proteases. ADAM proteins are type I transmembrane glycoproteins composed of several functional domains: a prodomain (maintains latency), a metalloproteinase domain (catalyzes substrate cleavage), a disintegrin domain (mediates cell adhesion), a cysteine-rich domain (involved in substrate recognition and dimerization), an EGF-like domain (may participate in protein–protein interactions; note that ADAM10 and ADAM17 lack an EGF-like domain), a transmembrane domain (anchors the protein), and a cytoplasmic tail (regulates intracellular signaling and trafficking). After activation, ADAM proteases regulate key cellular processes, including signaling, adhesion, migration, and inflammation through ectodomain shedding of membrane-bound substrates (e.g., cytokines, growth factors, and receptors). Note: Arrows indicate downstream effects; dashed lines represent cleavage by ADAM proteases. Created by Biorender. Yufei Chen, Dong Xiang. (2025) https://www.biorender.com/ (accessed on 4 December 2025).

The structure of ADAM proteins comprises the prodomain, metalloproteinase domain, disintegrin domain, cysteine-rich domain, EGF-like domain, transmembrane domain, and cytoplasmic tail domain (Figure 1). This modular architecture not only imparts unique molecular recognition and catalytic activities to ADAM proteins but also allows for the precise regulation of a diverse array of biological processes, including cell adhesion, signal transduction, and proteolysis, through the functional specialization of their distinct domains.

#### 2.1.1. Prodomain

The prodomain of ADAM proteins, consisting of 158 to 174 amino acids, contains a conserved cysteine residue that occupies the active site before activation. Following biosynthesis, ADAMs are transported to the endoplasmic reticulum as inactive precursors. Their prodomains are subsequently cleaved in the trans-Golgi network via either autocatalytic processing or proprotein convertases. Additionally, the prodomain acts as a molecular chaperone, facilitating the proper transport of mature ADAM proteins to the cell surface via the secretory pathway [1]. The primary role of this domain is to maintain the protease in an inactive zymogen state. Its removal through cleavage triggers the activation of ADAM’s catalytic function.

#### 2.1.2. Metalloproteinase Domain

The metalloproteinase domain adopts a globular structure, characterized by a highly conserved HExxHxxGxxH sequence at its active site, which is the core of its catalytic activity and constructs a zinc ion-dependent hydrolysis center. The three histidine (H) residues directly chelate the essential catalytic zinc ion (Zn^2+^), forming a stable metal ion coordination structure. Meanwhile, the glutamic acid (E) residue acts as the catalytic base, responsible for activating and polarizing the water molecule bound to the zinc ion, thereby converting it into a highly reactive nucleophile (OH^−^) [8]. This domain is present in all ADAM proteins; however, only approximately half of them retain catalytic activity [9,10,11]. When the extracellular stem region of the substrate transmembrane protein enters the active center, the binding pocket formed by this sequence recognizes and precisely locates the substrate, making the peptide bond to be cleaved adjacent to the catalytic zinc ion [12]. Subsequently, the activated hydroxyl ions launch a nucleophilic attack on the carbonyl carbon of the peptide bond. Zn^2+^ mediates this process by stabilizing the negatively charged transition state [13], ultimately hydrolyzing and breaking the peptide bond, thereby achieving the shedding and release of extracellular functional domains from the cell membrane.

#### 2.1.3. Disintegrin Domain

The disintegrin domain exhibits high homology with the core functional region of snake venom disintegrin. It typically consists of approximately 90 amino acids and facilitates binding to integrin receptors on the cell surface. The disintegrin-like domains in snake venom rely on their signature “RGD (Arg-Gly-Asp)” sequence for cell adhesion. In contrast, most members of the ADAM protein family cannot mediate adhesion in this way because they lack the RGD motif, except human ADAM15 [1,2,9]. As demonstrated by White et al., the consensus motif for integrin interaction in ADAM extracellular domains is CRXXXXXCDXXEXC [14]. Furthermore, another notable similarity between the disintegrin domains of ADAMs and their snake venom counterparts lies in the presence of disulfide bonds, which are crucial for maintaining structural rigidity [1]. The primary function of this domain is to regulate cell adhesion, migration, and interactions.

#### 2.1.4. Cysteine-Rich Domain

The cysteine-rich domain, which comprises 80–150 amino acids, is characterized by a high cysteine content but exhibits poor sequence conservation across this region [1]. This domain possesses diverse functional capabilities. It works with the metalloproteinase domain to identify and bind specific substrates. This teamwork enhances both the precision of binding and the efficiency of cleavage, as demonstrated by ADAM10’s action on the Ephrin-A5/Ephrin receptor A3 (EphA3) complex in human HEK293 cells [15]. It also enables ADAM proteins to form dimers via disulfide bonds or hydrophobic interactions. This dimerization is a key mechanism for regulating their catalytic activity. For example, ADAM8 self-assembles via its cysteine-rich region, thereby modulating its own activity [16]. Additionally, this domain contributes to cellular signal transduction processes.

#### 2.1.5. EGF-like Domain

The EGF-like domain consists of approximately 30 to 40 amino acids and contains six cysteine residues that form three disulfide bonds. Some variants of this domain exhibit repetitive sequences capable of accepting O-glycan modifications [10]. This domain is potentially involved in mediating cell–cell interactions and functions as a storage reservoir for EGF.

#### 2.1.6. Transmembrane Domain and Cytoplasmic Tail Domain

The transmembrane and cytoplasmic tail domains exhibit significant variability in both length and sequence among members of the ADAM family. Traditionally, the transmembrane domain serves primarily as a membrane anchor, ensuring proper cellular localization. The cytoplasmic tail domain is distinguished by its high proline content and low sequence conservation. It facilitates bidirectional signal transduction between extracellular and intracellular compartments and acts as a ligand for SH3 protein domains, thereby contributing to intracellular signaling pathways [17].

The ADAM protease family achieves functional integration through its modular domains (prodomain, metalloprotease domain, disintegrin domain, cysteine-rich domain, EGF-like domain, transmembrane domain, and cytoplasmic tail), enabling it to simultaneously perform protein hydrolysis and cell adhesion tasks while bidirectionally integrating intracellular signaling with the extracellular environment [18]. This multifunctional domain configuration profoundly influences processes critical to cell fate determination (e.g., Notch signaling), inflammatory responses (e.g., TNF-α shedding), cancer progression (e.g., growth factor receptor activation and cell migration), and fertilization. These processes critically depend on the precise, localized integration of proteolytic cleavage, and cell adhesion [18]. The mechanism by which ADAM proteases target distinct substrates is multifaceted, relying not only on the catalytic activity of the metalloprotease domain but also involving other extracellular domains for substrate recognition, cytoplasmic tail-mediated subcellular localization and activity regulation, conformational features of the substrate’s membrane-proximal sequence, and cell type-specific expression and signaling pathway activation. Together, these layers confer specificity and regulatability to substrate selection [18].

### 2.2. Regulation of ADAM Protease Activation

The activation of ADAMs is a complex process regulated by multiple synergistic mechanisms [3,19], with its protein expression levels being influenced at transcriptional, translational, and post-translational levels. At the transcriptional level, ADAM family genes are coordinately regulated by transcription factors (e.g., NF-κB, SP1), epigenetic modifications (e.g., DNA methylation, histone acetylation), cellular signaling pathways (e.g., TNF-α, JNK), and microenvironmental signals (e.g., hypoxia, inflammation) [20,21,22,23,24,25], thereby enhancing ADAM transcriptional expression. Following translation, ADAM proteins reside as inactive precursors, with their functional fate critically dependent on subsequent post-translational modifications, thereby conferring dynamic and context-dependent regulation of their active expression in various physiological and pathological processes [26,27].

ADAMs are synthesized in the endoplasmic reticulum (ER) as an inactive proenzyme. During transport through the Golgi apparatus, the conserved RX (R/K) R motif in the pre-prodomain can be released from inhibition through cleavage by proteases such as furin protease, and some members (e.g., ADAM8) can even complete the removal of the pre-prodomain through autocatalysis [3,19]. However, prodomain cleavage alone is not sufficient to achieve full activation; ADAM proteases also require binding to specific cofactors. ADAM proteases are further regulated through specific protein interactions and post-translational modifications. For instance, the C8 family of transmembrane proteins regulates the membrane localization and substrate selectivity of ADAM10, while inactive rhomboid proteins (iRhoms) are responsible for the trafficking and maturation of ADAM17 [3,19]. Additionally, phosphorylation mediated by kinases such as MAPK (ERK, p38) or protein kinase C (PKC) can enhance ADAM activity by releasing autoinhibitory conformations and inducing dimer dissociation into active monomers [3,19]. After activation, ADAMs need to be targeted to specific subcellular regions, such as the cell membrane or endosomes, through intracellular sorting signals and transport mediated by accessory proteins before they can bind to membrane-anchored substrates and perform cleavage function [3,19]. The activation of ADAM proteases is further refined by multiple regulatory mechanisms. These include trafficking controls such as ER retention and endosomal processing; inhibition by endogenous proteins like TIMP3, which binds to the catalytic domain to block activity; and substrate-intrinsic regulation, where the intracellular domain of certain substrates (e.g., L-selectin) can sterically hinder cleavage [3,19]. As core members, ADAM10 and ADAM17 exhibit specific activation patterns. ADAM10 relies on the TspanC8 family for substrate selectivity regulation, while ADAM17 is regulated by iRhoms for mature transport. These two enzymes activate at distinct sites—the plasma membrane and endosomes, respectively—to cleave different critical substrates [3,19].

### 2.3. Comparative Analysis of Structural Features and Expression Patterns of Major ADAM Proteases

The ADAM family, characterized by its multi-domain structure, is extensively involved in various physiological and pathological processes, including extracellular matrix remodeling, cell adhesion, and signaling pathway regulation [28]. Among the numerous ADAM members, ADAM10, ADAM17, ADAM9, and ADAM12 have attracted considerable attention due to their critical roles in regulating tissue homeostasis and in the pathogenesis of various diseases [28,29].

#### 2.3.1. Structural Differences in the Main Proteinases of ADAM

ADAM10 and ADAM17 are two catalytically active members of the ADAM family that are functionally critical and highly structurally homologous. Their most notable structural similarity is the absence of the EGF-like domain, a core feature distinguishing them from other majority members such as ADAM9 and ADAM12 [1]. The metalloprotease domains of both ADAM10 and ADAM17 contain a conserved zinc-binding motif essential for their catalytic activity [1]. Despite structural similarities, they exhibit distinct substrate selectivities, primarily determined by differences in the depth of their prime S1’ pockets. ADAM10 possesses a deeper S1’ pocket that accommodates bulky aromatic residues, while ADAM17 has a shallower pocket that prefers small hydrophobic residues [12]. The intracellular trafficking, membrane localization, and functional activity of ADAM10 depend on interactions with proteins from the Tetraspanin C8 family [30]. In contrast, the maturation, transport, and activity of ADAM17 strictly require the assistance of inactive rhomboid proteins (iRhom1 and iRhom2) [1,31]. Furthermore, their N-terminal domains not only inhibit enzyme activity through the “cysteine switch” mechanism but also act as molecular chaperones to ensure the correct folding and transport of proteins. Specific mutations in the prodomain of ADAM10 (Q170H, R181G) impair its maturation process [32]. Recent structural biology studies have further revealed that the full extracellular domain of ADAM10 adopts a unique conformation in which its cysteine-rich (C) domain is positioned close to the catalytic cleft, thereby exerting self-inhibition. Functional evidence suggests that ADAM17 likely employs a similar C-domain-mediated self-inhibition mechanism, providing a structural basis for its rapid activity upregulation upon cellular stimulation [1].

Unlike ADAM10 and ADAM17, ADAM9 and ADAM12 feature the canonical multi-domain architecture typical of the ADAM protease family [1,5]. Therefore, the presence of the EGF-like domain is what distinguishes their core structure from the former two [1]. A shared structural feature is that both can generate secreted variants through alternative splicing, such as ADAM9-S and ADAM12-S. These variants lack transmembrane domains and cytoplasmic tails, enabling their release into the extracellular space to exert their functions [1]. Functionally, both exhibit overlapping and distinct characteristics. They share the ability to cleave several common substrates, such as ligands of the EGF receptor (EGFR). Meanwhile, ADAM12 has drawn particular attention in muscle development and tumor progression due to its capacity to cleave insulin-like growth factor binding proteins (IGFBPs) [5]. In terms of cell interactions, the disintegrin domain of ADAM9 can bind to multiple integrins (e.g., α6β1, αvβ5) to mediate cell adhesion [5], while ADAM12 plays a crucial role in myoblast fusion [31]. An important biochemical characteristic difference lies in their sensitivity to tissue metalloproteinase inhibitors (TIMPs). ADAM9 is not inhibited by TIMPs [5], while ADAM12 is inhibited by TIMP2 and TIMP3 [5].

#### 2.3.2. Differences in the Expression Patterns of the Main ADAM Proteases

As important members of the ADAM family, ADAM10, ADAM17, ADAM9, and ADAM12 exhibit tissue-specific expression patterns and are closely related to physiological and pathological processes. ADAM10 demonstrates widespread expression, being highly expressed in fibroblasts and endothelial cells during embryogenesis and participating in embryonic developmental regulation [33], and persisting in the brain, cardiovascular system, and epithelial tissues during adulthood [34]. Its expression is significantly increased in the myocardial tissues of patients with dilated cardiomyopathy and atrial fibrillation [33], and it is also upregulated during the invasion of tumor cells into surrounding tissues [34]. ADAM17 is more ubiquitously expressed, covering almost all embryonic tissues and parenchymal cells and immune cells of adult tissues [35]. It is highly expressed throughout the embryonic period and is crucial for development. In adulthood, it maintains a relatively high level in immune tissues and cardiovascular systems, with significant upregulation during pathological processes such as inflammatory responses, myocardial infarction, and atherosclerosis [33,36]. Moreover, its expression is also increased in the colonic mucosa of patients with inflammatory bowel disease [33]. ADAM9 expression exhibits considerable breadth, involving muscle cells, brain, epithelial tissues, and endothelial cells [33]. After adulthood, its expression is particularly prominent in cardiac muscle tissue, skeletal muscle, and brain tissue. Polymorphisms in its promoter region are associated with the susceptibility to sporadic Alzheimer’s disease [33,34]. Although it participates in invasion-related processes in tumor tissues, the amplitude of its expression change is not as significant as that of ADAM10 and ADAM17 [33]. Unlike the three proteases mentioned above, ADAM12 expression is comparatively restricted, mainly localized to the placenta, cardiomyocytes, smooth muscle cells, and reproductive tissues [33]. It participates in myogenesis during embryogenesis and exhibits prominent functions in the cardiovascular system during adulthood. Its expression is upregulated in myocardial tissue from patients with hypertrophic cardiomyopathy, in pathological conditions involving vascular smooth muscle cell proliferation [33,37], and in myocardial tissues from heart failure models [38].

### 2.4. Substrates of ADAM Protease

Ectodomain shedding is a proteolytic process in which the extracellular domain of a membrane protein is cleaved and released as a soluble fragment, while a membrane-bound remnant is retained. This process regulates protein function through activation or inactivation and participates in essential cellular processes, including signal transduction, adhesion, proliferation, and differentiation. The ectodomain undergoes proteolytic cleavage in response to specific stimuli, generating functionally diverse proteins encompassing cytokines, growth factors, cell surface receptors, and adhesion molecules [6,39,40]. Ectodomain shedding is principally mediated by zinc-dependent metalloproteinases. Variations in protease domain specificity enable distinct family members to recognize and cleave different substrate types (Table 1). ADAM10 and ADAM17 are two key proteases within the ADAM family. Proteolytic processing of these substrates contributes to physiological processes spanning embryonic development, immune responses, and tissue repair, while its dysregulation is closely associated with various pathological conditions [41].

#### 2.4.1. Cytokines

Cytokines are multifunctional signaling proteins transiently secreted by activated cells. Their functions include regulating immune responses, cell proliferation, differentiation, and apoptosis, thereby playing important roles in processes such as inflammation, tumor development, and tissue repair [42,43]. Ectodomain shedding mediated by ADAM proteases represents one of the key regulatory mechanisms controlling cytokine activity. This proteolytic cleavage converts membrane-anchored precursors into soluble, biologically active forms, thereby modulating their signaling range, potency, and function.

By cleaving inflammatory factors (e.g., TNF-α) and chemokines (e.g., CX3CL1, CXCL16), ADAM family proteases amplify inflammatory signals and modulate the recruitment of immune cells. In 1997, Black et al. first identified ADAM17 as the TNF-α-converting enzyme (TACE) and established its essential role in cleaving the membrane-bound TNF-α precursor to release biologically active soluble TNF-α [44]. Then, soluble TNF-α regulates the inflammatory response of macrophages, apoptosis, and necrosis by modulating the expression of the cholesterol transporter ATP-binding cassette transporter A1(ABCA1), the TRAF3-TAK1-MAPK axis, as well as death receptors DR3, TRAIL, and Fas [45,46,47]. ADAM10 and ADAM17 proteolytically process the membrane-bound CX3CL1, generating soluble CX3CL1, which functions as a chemoattractant for monocytes, T cells, and NK cells by activating the CX3CR1 pathway [48]. It further modulates key signaling pathways such as p38 MAPK and NF-κB [48,49], precisely regulating microglial activity and neuroinflammation levels. By cleaving CXCL16 on the endothelial lumen surface and in the subendothelial space, ADAM10 regulates the adhesion and migration of key immune cells, including activated T cells and macrophages, subsequently promoting the development of chronic inflammatory diseases such as atherosclerosis [50]. Thus, ADAM-mediated shedding serves as a critical switch for activating cytokines and chemokines, converting them from local membrane-bound signals into systemic or targeted soluble mediators.

#### 2.4.2. Growth Factors

Growth factors are signal peptides or proteins derived from a class of cells (such as platelets, nerve cells, immune cells, various epithelial cells and connective tissue cells), which function by binding to specific surface receptors, thereby activating the phosphorylation cascade reaction and coordinating necessary cellular processes, including proliferation, differentiation, migration, survival and metabolic regulation [51]. Many substrates classified as growth factors initially exist as inactive membrane-anchored precursors (pro-GFs). ADAM-mediated proteolysis liberates biologically active fragments, enabling them to trigger downstream signaling pathways [52].

ADAM family members regulate cell proliferation, differentiation, or damage repair in diseases by cleaving growth factors mainly composed of EGF family ligands, such as transforming growth factor-alpha (TGF-α), EGF, heparin-binding epidermal growth factor-like growth factor (HB-EGF), amphiregulin (AREG), epiregulin, and betacellulin [53]. By utilizing ADAM17 knockout mouse models, Peschon et al. identified ADAM17 as the principal sheddase responsible for TGF-α cleavage, an event that releases soluble ligands to activate the EGFR signaling pathway [35], thus activating the ERK signaling pathway, which leads to cell proliferation [54]. ADAM17 can also splice HB-EGF, a process regulated by the signaling pathway composed of protein kinase C alpha (PKC-α) and protein phosphatase 1 regulatory subunit 14D (PPP1R14D), and is involved in the occurrence and development of various diseases such as myocardial hypertrophy, breast cancer, and kidney diseases [55]. ADAM17 hydrolyzes and cleaves the transmembrane precursor protein AREG, generating and releasing biologically active soluble AREG, which in turn activates downstream MAPK and phosphoinositide 3-kinase (PI3K) signaling pathways, precisely regulating key processes such as breast duct development and skin wound healing under physiological conditions [56,57]. In addition, ADAM17 cleaves the membrane-bound precursor Epiregulin, generating a biologically active soluble mature Epiregulin. This regulates downstream EGFR homodimers or heterodimers (such as EGFR/erythroblastic leukemia viral oncogene homolog 2 (ErbB2)) and their corresponding MEK/ERK and PI3K/AKT signaling pathways. This process regulates proliferation, differentiation, and injury repair in human airway epithelial cells, while under pathological conditions, it drives tumorigenesis, epithelial–mesenchymal transition, angiogenesis, and immune evasion in non-small-cell lung cancer [58].

As the key sheddase for membrane-bound EGF precursors, ADAM10 processes the juxtamembrane region to generate soluble EGF, which regulates the EGFR signaling pathway [59] and further affects the ERK1/2 signaling pathway [59]. ADAM10 can also activate the epidermal growth factor receptor signaling pathway by shedding Betacellulin to produce soluble Betacellulin, which is specifically manifested as effectively inducing the phosphorylation of downstream ERK1/2 only in the presence of ADAM10 [59].

#### 2.4.3. Cell Surface Receptors

As integral membrane proteins with high binding affinity, cell surface receptors recognize diverse extracellular ligands encompassing neurotransmitters, hormones, and growth factors, execute conformational rearrangements, and transduce signals across the membrane [60]. It mainly includes ion channel-coupled receptors, G protein-coupled receptors, and enzyme-linked receptors, which can participate in hormone regulation, promote signal transmission, and influence cell growth, differentiation, and metabolism [60]. By generating soluble receptor variants through ectodomain shedding, ADAM proteases exert dual control over signaling pathways, capable of both potentiation and suppression.

The ADAM family cleaves enzyme-linked receptors (e.g., ACE2, IL-6R, c-Met, and Notch), c-type lectin superfamily (e.g., CD23), and tumor necrosis factor receptor superfamily (e.g., CD30), which regulate physiological and pathological processes. Using HEK293 and Huh7 cell lines, Lambert and colleagues demonstrated that ADAM17 catalyzes the regulated ectodomain shedding of membrane-anchored angiotensin-converting enzyme 2 (ACE2), yielding soluble ACE2 (sACE2) [61]. This further affects the occurrence and development of neurogenic hypertension, the enhancement of sympathetic nerve excitability, the pathological phenotypic transformation of vascular smooth muscle cells, and vascular remodeling, among other pathological processes of the cardiovascular and nervous systems [62,63,64]. ADAM10 and ADAM17 cleave IL-6R to generate soluble receptors that mediate the IL-6 trans-signaling, jointly regulating the inflammatory and immune responses of the body [65,66]. Additionally, ADAM10 and ADAM17 cleave the hepatocyte growth factor (HGF) receptor c-Met to produce its soluble form sMet, which can competitively bind to HGF and negatively regulate the intensity of the HGF/c-Met signaling pathway, thereby affecting the repair and regeneration process after liver injury [67,68]. ADAM10 cleaves the Notch receptor to generate the Notch intracellular domain (NICD), which regulates the expression of downstream genes such as Hairy and enhancer of split 5 (*Hes5*) and delta-like 1 (*Dll1*), thereby influencing embryonic development (including the formation of the nervous system, somites, and cardiovascular system). Its deficiency leads to embryonic lethality. ADAM17 also participates in the cleavage of the Notch extracellular domain [69].

ADAM10 modulates pathological immune responses by cleaving the membrane-bound low-affinity IgE receptor (mCD23) on B cell surfaces. This cleavage releases soluble CD23 (sCD23), which binds to CD21, driving B cell activation and IgE synthesis, thereby amplifying IgE-mediated allergic disorders such as allergic rhinitis and atopic dermatitis [70]. ADAM17 generates soluble CD30 by cleaving membrane-bound CD30. This soluble form subsequently modulates the Th1/Th2 immune balance by antagonizing membrane CD30 signaling and inhibiting Th1-type cytokines. Thus, it influences pathological processes in Th1-dominant diseases, including Hodgkin’s lymphoma and autoimmune disorders such as multiple sclerosis and rheumatoid arthritis [71].

#### 2.4.4. Cell Adhesion Molecules

Cell adhesion molecules represent a specialized class of cell-surface glycoproteins responsible for specific intercellular and cell–matrix interactions. Their extracellular domains achieve precise adhesion through homophilic binding with identical molecules and heterophilic engagement with complementary ligands [72]. These molecules are classified into two major categories, namely calcium-dependent (e.g., cadherins and selectins) and calcium-independent (e.g., the immunoglobulin superfamily and integrins) [72]. The ADAM protease family modulates intercellular interactions by cleaving specific adhesion molecules, thereby affecting tissue morphogenesis and remodeling under pathological conditions.

ADAM family proteases target cell adhesion molecules, including E-cadherin, L-selectin, vascular cell adhesion molecule-1 (VCAM-1), and CD44, and play roles in embryonic development, immunity, inflammation, and cancer metastasis. ADAM10 cleaves E-cadherin to generate soluble E-cadherin fragments and a membrane-bound C-terminal fragment, thereby regulating the subcellular localization of β-catenin and its downstream signaling pathways (Cyclin D1). This influences epithelial cell adhesion, migration, proliferation, and embryonic development, and may contribute to tumor progression [73]. ADAM17 cleaves L-selectin on the surface of immune cells (neutrophils and T cells), generating its soluble form and potentially liberating its transmembrane fragment. Consequently, it modulates signaling pathways mediated by factors like redox status, PKC, and IL-2, ultimately influencing the intensity of inflammatory responses and the efficacy of T-cell-mediated anti-viral immune responses [74,75].

In a cell model stimulated by phorbol 12-myristate 13-acetate (PMA), Garton et al. discovered that ADAM17 mediated proteolytic cleavage of VCAM-1, leading to the shedding of its extracellular domain and the generation of soluble VCAM-1 (sVCAM-1) fragments [76]. sVCAM-1 acts as a chemoattractant and inflammatory activator, which through its binding to the α4β1 integrin receptor, recruits neutrophils and activates alveolar macrophages, thereby influencing the process of pathological neutrophil infiltration and amplification of inflammation in acute lung injury/acute respiratory distress syndrome [77]. ADAM10 cleaved the extracellular domain of the CD44 glycoprotein on the melanoma cell membrane, generating soluble CD44 fragments. By regulating the hyaluronic acid (HA)-CD44 signaling pathway and its downstream ezrin, radixin, moesin (ERM)/neurofibromin 2 (NF2) complex, it affected the malignant proliferation behavior of melanoma. [78].

#### 2.4.5. Others

In addition to the above-mentioned substrates, ADAM proteases can cleave numerous other disease-associated proteins such as insulin-like growth factor binding protein-3 (IGFBP-3), cellular prion protein (PrP^C^), and amyloid precursor protein (APP), highlighting their multifaceted regulatory roles. ADAM28 cleaves IGFBP-3, generating cleaved IGFBP-3 fragments and releasing free, biologically active insulin-like growth factor-1 (IGF-1). This IGF-1 then modulates PI3K/AKT/mTOR and rat sarcoma virus oncogene homolog (RAS)/MAPK signaling pathways through the IGF-1 receptor (IGF-1R), thereby influencing tumor cell proliferation, survival, metastasis, and chemotherapy resistance [79,80]. ADAM10 cleaves full-length PrP^C^ on the cell membrane, generating soluble PrP^C^ (sPrP^C^). This process modulates PrP^C^-mediated neurotoxic signaling, delays the progression of prion diseases, alleviates amyloid-β(Aβ)-induced neurotoxicity in Alzheimer’s disease, and may exert neuroprotective and regenerative effects under pathological conditions such as stroke [81]. ADAM9, ADAM10, and ADAM17 regulate the Aβ peptide generation pathway by cleaving APP to produce soluble APPα fragments and C83 fragments, and influencing the pathological process of Alzheimer’s disease [82].

In summary, various substrates of the ADAM proteases, including cytokines, growth factors, cell surface receptors, and adhesion molecules, are collectively regulated by the key post-translational modification mechanism of ADAM-mediated extracellular domain shedding. This cleavage event, determined by proteolysis, modulates protein function. For cytokines and growth factors, shedding serves as the critical step that converts inactive precursors into active, soluble signaling molecules, thereby enabling functional activation. For receptors and adhesion molecules, shedding provides a dynamic regulatory mechanism. By generating soluble receptor variants or adhesion molecule fragments, it precisely modulates downstream signaling networks and cellular behaviors through agonistic, antagonistic, or chemotactic effects. Consequently, ADAM-mediated shedding represents a vital pathway for substrate functionalization. The released soluble extracellular domain substrates act as active signal modulators, extensively participating in the modulation of both physiological and pathological processes.

**Table 1:** Substrates of ADAM Proteases and Their Biological Functions.

Category	Subcategory	Substrates	ADAMs	Biological Function	Types of Diseases	Types of Study	References
Cytokines	Inflammatory factors	TNF-α	ADAM17	Inflammation and immunity regulation	Inflammatory diseases	In vitro	[44]
	Chemokines	CX3CL1	ADAM17 ADAM10	Immune cell migration and inflammatory response	Traumatic brain injury and spinal cord injury	In vivo and in vitro	[48]
		CXCL16	ADAM10	T cell migration and immune response	Vascular inflammatory disease	In vitro	[50]
Growth factors	EGF family ligands	TGF-α	ADAM17	Cell proliferation and tumor growth	Developmental abnormalities	In vivo and in vitro	[35]
		EGF	ADAM10	Cell growth and differentiation	Cancer	In vitro	[59]
		HB-EGF	ADAM17	Cell proliferation and migration	Cardiac hypertrophy	In vitro	[55]
		Amphiregulin	ADAM17	Cell proliferation and differentiation	Mammary gland development, breast cancer, hyperproliferative skin diseases, and wound healing	In vivo and in vitro	[56,57]
		Epiregulin	ADAM17	Cell proliferation, differentiation, and migration	Non-small-cell lung cancer	In vivo and in vitro	[58]
		Betacellulin	ADAM10	Cell proliferation, differentiation, and apoptosis	Functional verification of Betacellulin	In vivo and in vitro	[59]
Cell surface receptors	Enzyme-linked receptor	ACE2	ADAM17	Antihypertensive, antifibrotic, and antiviral effects	Severe acute respiratory syndrome	In vitro	[61]
		IL-6R	ADAM17 ADAM10	Immune cell activation and inflammatory signaling	Inflammatory diseases and colon cancer	In vivo and in vitro	[65,66]
		c-Met	ADAM10 ADAM17	Hepatocyte proliferation, migration, and regeneration	Liver diseases	In vivo and in vitro	[67,68]
		Notch	ADAM10 ADAM17	Regulate the development of the nervous system and cardiovascular system, and control the generation of blood cells and blood vessels	Alzheimer’s disease	In vivo and in vitro	[69]
	C-type lectin superfamily	CD23	ADAM10	B-cell proliferation and macrophage activation	Allergic diseases	In vivo and in vitro	[70]
	Tumor necrosis factor receptor superfamily	CD30	ADAM10	T-cell and B-cell proliferation	Lymphoma, autoimmune and inflammatory diseases, infectious and allergic diseases	In vitro	[71]
Cell adhesion molecules	Cadherins	E-cadherin	ADAM10	Tumor metastasis promotion	Tumors	In vitro	[73]
	Selectins	L-selectin	ADAM17	Regulate the immune response	Inflammatory diseases and viral infections	In vivo and in vitro	[74,75]
	The immunoglobulin superfamily	VCAM-1	ADAM17	Inflammation and immune response	Inflammatory diseases	In vitro	[76]
	Others	CD44	ADAM10	Inflammation regulation and tumor metastasis promotion	Melanoma	In vivo and in vitro	[78]
Others	Disease-related factors	IGFBP-3	ADAM28	Cell proliferation, migration, and tumor progression	Malignant tumors	In vivo and in vitro	[79,80]
		PrPc	ADAM10	Neuroprotection	Neurodegenerative diseases	In vivo, in vitro, and preliminary clinical analysis	[81]
		APP	ADAM9 ADAM10 ADAM17	Alzheimer’s disease Pathogenesis	Alzheimer’s disease	In vitro and clinical analysis	[82]

## 3. Role of ADAM Proteases in Liver Pathologies

**Figure 2:**
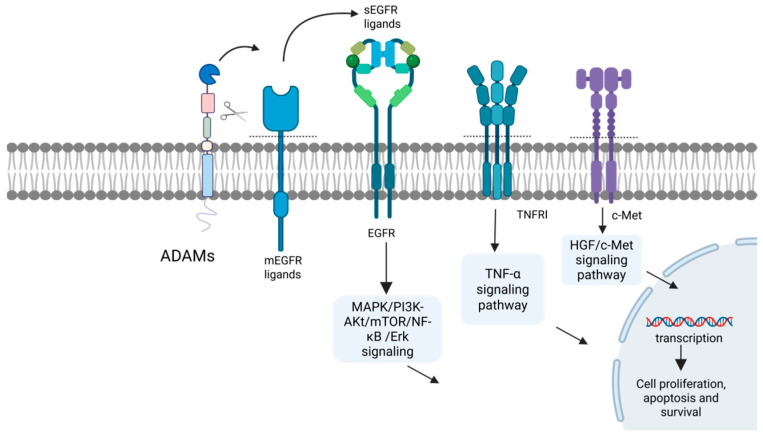
Regulation of hepatocyte injury and death by ADAM proteases. ADAM family members modulate hepatocyte fate through proteolytic processing of membrane-tethered ligands and receptors. For instance, ADAM17 cleaves precursors of EGFR ligands (e.g., TGF-α, HB-EGF), releasing soluble forms that activate EGFR and downstream pro-survival pathways (MAPK/ERK, PI3K/Akt). ADAM10 and ADAM17 can also shed the c-Met receptor, attenuating HGF/c-Met signaling. Meanwhile, ADAM-mediated release of TNF-α and cleavage of TNFR1 influence apoptotic and inflammatory responses. The balance of these activities determines hepatocyte survival, death, and regenerative capacity in various liver injury contexts. Note: Arrows indicate downstream signaling pathways; dashed lines represent cleavage by ADAM proteases. Created by Biorender. Yufei Chen, Xuepeng Gong. (2025) https://www.biorender.com/ (accessed on 4 December 2025).

**Figure 3:**
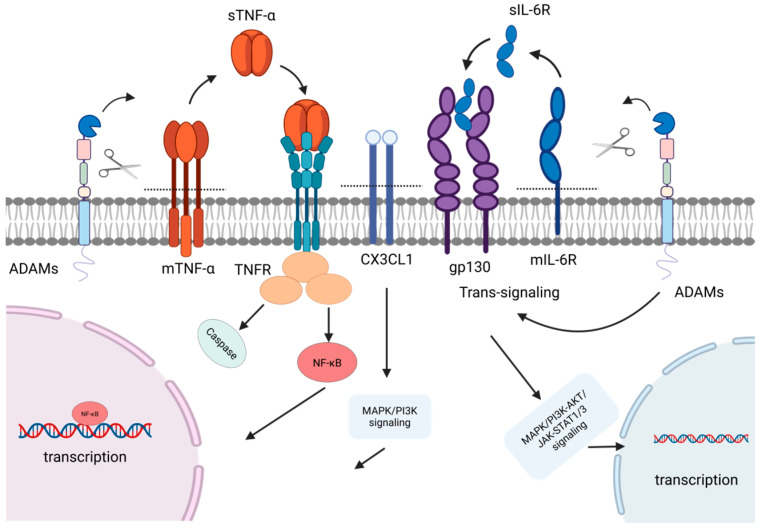
Modulation of liver inflammation by ADAM proteases. ADAM proteases, particularly ADAM17 and ADAM10, amplify hepatic inflammatory responses by shedding key membrane-associated mediators. Cleavage of membrane-bound TNF-α by ADAM17 releases soluble TNF-α (sTNF-α), which activates TNFR1 on target cells and drives pro-inflammatory signaling (NF-κB, MAPK). ADAM17 and ADAM10 also shed the IL-6 receptor (IL-6R), generating soluble IL-6R (sIL-6R) that combines with IL-6 to activate gp130-mediated trans-signaling in cells lacking membrane IL-6R. Additionally, ADAM-mediated cleavage of chemokines such as CX3CL1 regulates leukocyte recruitment and inflammatory cascades. Note: Arrows indicate downstream signaling pathways; dashed lines represent cleavage by ADAM proteases. Created by Biorender. Yufei Chen, Dong Xiang. (2025) https://www.biorender.com/ (accessed on 4 December 2025).

**Figure 4:**
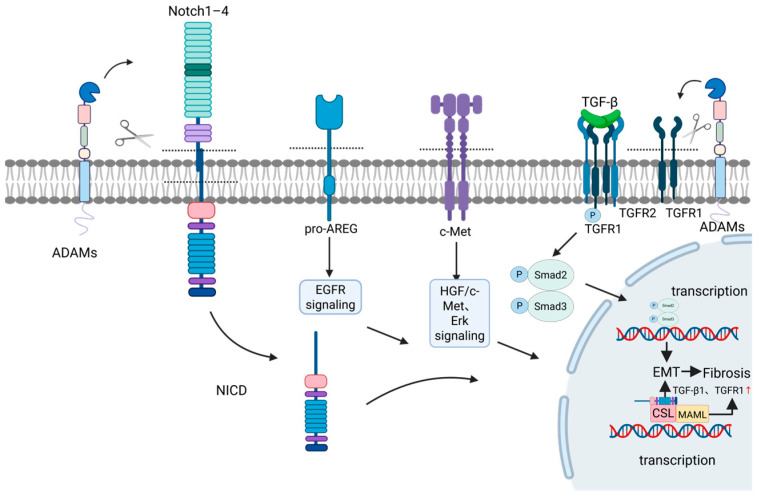
Role of ADAM proteases in hepatic stellate cell activation and liver fibrosis. ADAM proteases promote hepatic fibrosis by driving hepatic stellate cell (HSC) activation and extracellular matrix deposition. ADAM17 cleaves Notch receptors, releasing the Notch intracellular domain (NICD), which translocates to the nucleus and upregulates TGF-β and its receptors, amplifying fibrogenic signaling. ADAM17 also sheds pro-AREG, activating EGFR on HSCs. ADAM10 can negatively regulate HSC activation via c-Met shedding, while ADAM12 enhances TGF-β signaling and integrin-linked kinase (ILK) activity, promoting HSC survival and fibrotic responses. Note: Arrows indicate downstream signaling pathways; dashed lines represent cleavage by ADAM proteases. Created by Biorender. Yufei Chen, Dong Xiang. (2025) https://www.biorender.com/ (accessed on 4 December 2025).

**Figure 5:**
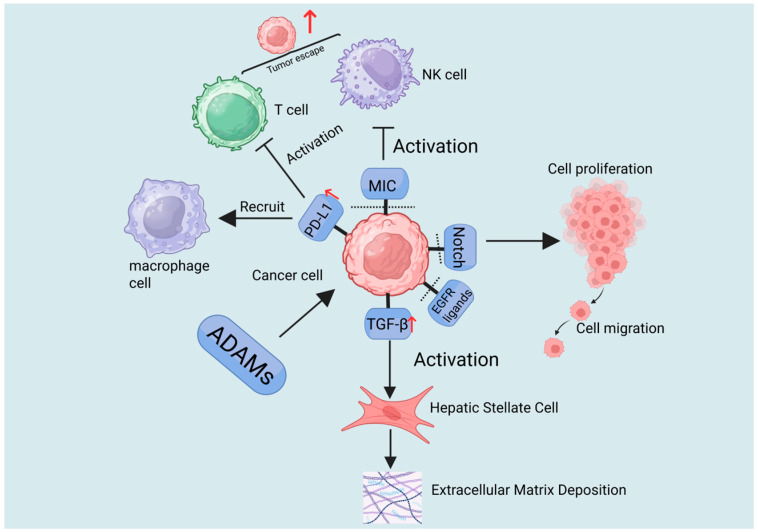
Mechanisms by which ADAM proteases drive hepatocellular carcinoma progression. ADAM family members contribute to HCC pathogenesis through multiple mechanisms: (1) Cleavage of EGFR ligands and Notch receptors activates oncogenic signaling pathways (EGFR, PI3K/Akt, Notch) in hepatoma cells, promoting proliferation, migration, and invasion. (2) Shedding of immune ligands such as MICA/B by ADAM9 reduces NK cell activation, while ADAM15-mediated PD-L1 release inhibits T cell function, facilitating immune escape. (3) ADAMs remodel the extracellular matrix and recruit tumor-associated macrophages, fostering a pro-tumorigenic and immunosuppressive microenvironment. Note: Arrows indicate downstream signaling pathways; dashed lines represent cleavage by ADAM proteases. Created by Biorender. Yufei Chen, Dong Xiang. (2025) https://www.biorender.com/ (accessed on 4 December 2025).

The liver serves as the central organ for metabolic and immune regulation in the body, making it susceptible to various factors such as viruses, toxins, and immune dysregulation, which can trigger diverse acute and chronic pathological changes [83]. The ADAM family of transmembrane metalloproteinases plays a pivotal role throughout the entire process of liver disease progression, spanning multiple stages from initial injury, inflammatory response, compensatory repair, fibrosis, to terminal malignant transformation [84]. This family primarily participates in multiple hepatic pathological processes by regulating cytokine release, activating signaling pathways, and mediating intercellular adhesion. Key mechanisms include regulating hepatocyte injury and death, modulating hepatic inflammatory responses, activating hepatic stellate cells (HSCs) to drive fibrogenesis, and propelling HCC pathogenesis (Table 2, Figure 2, Figure 3, Figure 4 and Figure 5) [85,86].

### 3.1. Regulation of Hepatocyte Injury and Death

Hepatocytes, the principal functional units of the liver, perform essential tasks including metabolism, synthesis, and detoxification. Their injury and death play a decisive role in the onset and progression of liver diseases, serving not only as key triggers for inflammatory responses and fibrosis initiation but also as major drivers propelling the disease toward advanced malignant transformation [87]. Across different types of liver diseases, ADAM proteases may participate in regulating hepatocyte injury and death through diverse mechanisms (Figure 2).

As a key protease, ADAM17 regulates hepatocyte injury and death in ways that vary depending on the etiology, stage, and cellular microenvironment of liver injury. In models of fulminant hepatitis induced by the Fas agonist Jo-2 or acetaminophen (APAP), ADAM17 plays a critical hepatoprotective role through its protein cleavage function [88]. ADAM17 cleaves and releases membrane-bound tumor necrosis factor receptor 1 (TNFR1), thereby reducing cellular responsiveness to TNF signaling and attenuating the activation of downstream pro-apoptotic pathways. This includes diminished phosphorylation of JNK, reduced NF-κB activation, and suppression of the caspase-8 and caspase-3 cleavage cascade, ultimately inhibiting hepatocyte apoptosis [88]. Conversely, ADAM17 also cleaves and activates membrane-tethered EGFR ligands, such as amphiregulin, releasing soluble ligands that bind to the epidermal EGFR. This binding activates the ERK1/2 signaling pathway. Activated ERK1/2 then promotes the expression of anti-apoptotic proteins, including myeloid cell leukemia 1 (Mcl-1) and B-cell lymphoma-extra large (Bcl-xL), while simultaneously suppressing the function of the pro-apoptotic protein Bcl-2-like protein 11 (Bim), thereby initiating a robust anti-apoptotic effect [88].

Although ADAM17 exerts hepatoprotective effects under specific conditions, studies indicate it may promote inflammation and hepatocyte death in cholestatic liver disease. In a mouse model induced by bile duct ligation (BDL), hepatic ADAM17 expression and activity significantly increased [89]. After inhibiting ADAM17 with the specific inhibitor DPC 333, liver injury in BDL mice was mitigated, manifested by reduced serum ALT levels, decreased hepatic necrosis, and alleviated bile duct proliferation. Concurrently, ADAM17 inhibition alleviated sickness behaviors in BDL mice, characterized by improved social interaction and reduced immobility [89]. Furthermore, ADAM17 expression was markedly elevated in liver tissues from patients with primary biliary cholangitis (PBC) and primary sclerosing cholangitis (PSC) compared to healthy controls, particularly in hepatocytes, cholangiocytes, and immune cells (e.g., T cells and macrophages) [89]. These findings highlight the dual and context-dependent role of ADAM17 in hepatocyte injury, which is determined by the etiology and cellular microenvironment.

Other ADAM members also exert distinct effects on hepatocyte injury and death. ADAM10 serves as a core regulator of hepatic homeostasis, not only protecting hepatocytes to prevent the initiation of injury, but also precisely regulating the proliferation and differentiation of liver progenitor cells to maintain the order and balance of the regeneration process [90]. In a hepatocyte-specific ADAM10 knockout mouse model, the loss of ADAM10 transcriptionally downregulated the expression of bile acid transporters such as multidrug resistance-associated protein 2 (Mrp2) and bile salt export pump (BSEP), disrupting bile acid homeostasis and thereby directly triggering cholestasis, spontaneous hepatocyte necrosis, and liver fibrosis [90]. This persistent injury activated the liver regeneration program, leading to the activation of liver progenitor cells. Notably, ADAM10 itself negatively regulates the activity of the HGF/c-Met signaling pathway by cleaving the c-Met receptor ectodomain, thereby suppressing the excessive proliferation of liver progenitor cells. Meanwhile, in the fate determination of liver progenitor cell differentiation, ADAM10 moderately inhibits their differentiation toward hepatocytes to ensure normal biliary differentiation and maintain the architectural balance of the liver [90]. However, ADAM8 plays the opposite role in regulating the fate of liver cells. In a mouse model of acute liver injury induced by CCl4, intervention with anti-ADAM8 monoclonal antibodies found that the expression of vascular endothelial growth factor (VEGF), cytochrome P450 1A2 (CYP1A2), and proliferating cell nuclear antigen (PCNA) was significantly upregulated. This further promotes angiogenesis, metabolic function, and proliferation of liver cells in the liver, exerting therapeutic effects of liver protection and promoting repair [91]. ADAM9 exerts a dual regulatory role in liver injury, with its function varying depending on the pathological context. In a CCl4-induced liver injury model, pretreatment with an anti-ADAM9 monoclonal antibody resulted in more severe liver damage compared to mice that did not receive the antibody, as evidenced by increased serum ALT/AST levels, exacerbated tissue necrosis and inflammation, more significant hepatocyte apoptosis, reduced serum sIL-6R levels, accompanied by decreased expression of phosphorylated signal transducer and activator of transcription 3 (p-STAT3), PCNA, and VEGF proteins in liver tissue, as well as increased expression of Caspase3 and cytochrome P450 2E1 (CYP2E1) proteins [92]. In an alcohol-induced mouse model of acute liver injury, the *ADAM9* gene was specifically silenced by CRISPR/Cas9-sgRNA3. It was found that its deletion could significantly alleviate liver injury, manifested as a decrease in serum ALT/AST levels and a reduction in hepatocyte necrosis and apoptosis. Meanwhile, the expression of protective factors such as HSP27/HSP70, PCNA, B-cell lymphoma 2 (Bcl-2), VEGF, and p-STAT3 in liver tissue increased, while the expression of pro-apoptotic factors Bcl-2-associated X protein (Bax) and Caspase-3 decreased, indicating that ADAM9 has the effect of promoting hepatocyte injury and inhibiting regeneration in alcoholic liver injury [93]. The contradictory function of ADAM9 may be due to its ability to selectively cleave distinct membrane protein signals under different pathological stimuli, thereby triggering either protective or damaging signal cascade responses. However, the specific proteins that are cleaved and their downstream signals still require further investigation.

### 3.2. Modulation of Liver Inflammation

Inflammation is a cornerstone in the pathogenesis and progression of liver disease. Its chronic persistence significantly accelerates the transformation of liver injury to hepatitis and fibrosis, ultimately potentially leading to the development of HCC [94]. This pathological cascade initiates with the immune recognition of damage-associated molecular patterns (DAMPs) or pathogen-associated molecular patterns (PAMPs). Signaling pathways such as NF-κB propagate this response, culminating in the massive release of key pro-inflammatory cytokines like TNF-α and IL-1β [95]. As central regulators of hepatic inflammatory signaling, ADAM proteases, with ADAM17 playing a predominant role, modulate the intensity of the inflammatory cascade by cleaving membrane-bound mediators and receptors (Figure 3) [84].

ADAM17 regulates the proteolytic release of key pro-inflammatory factors, including TNF-α and IL-6R. This protease promotes soluble TNF-α (sTNF-α) production by cleaving its membrane-bound precursors on hepatic myeloid cells (e.g., Kupffer cells) and hepatocytes. The released sTNF-α then binds to TNFR1 on target cells, inducing receptor trimerization, recruitment of downstream signaling complexes, and activation of key pro-inflammatory signaling pathways such as NF-κB and MAPK. This cascade drives inflammatory responses and exacerbates liver injury [96]. Concurrently, ADAM17 cleaves membrane-bound TNFR1, releasing its soluble form (sTNFR1). Acting as a decoy receptor, sTNFR1 binds sTNF-α, preventing it from activating membrane-bound TNFR1, thereby providing a negative feedback mechanism that suppresses excessive TNF signaling and prevents uncontrolled inflammation. Under pathological conditions, the net effect of ADAM17 activity is to promote rather than suppress inflammation. Consequently, ADAM17 activity exacerbates the severity of inflammatory liver injury. Studies confirm that inhibiting ADAM17 alleviates both inflammation and tissue damage [96].

In liver diseases, ADAM17 serves as a key regulatory enzyme that directly influences IL-6 trans-signaling by mediating the release of soluble IL-6 receptor (sIL-6R) from its membrane-bound form, potentially making it a key contributor to the pro-inflammatory processes driven by IL-6 [97]. Specifically, when ADAM17 activity is inhibited or remains inactive, IL-6 primarily functions through the classical signaling pathway. By binding to the intact membrane-bound IL-6R (mIL-6R), it mainly activates limited cell types such as hepatocytes, thereby inducing STAT3 signaling to maintain liver homeostasis and support regenerative repair [98,99]. Conversely, when ADAM17 is activated and cleaves the mIL-6R, it initiates the IL-6 trans-signaling pathway, generating sIL-6R, which forms a complex with IL-6, thereby widely activating almost all cells expressing glycoprotein 130 (gp130) (particularly hepatic stellate cells and endothelial cells) [98]. This drives stronger and more persistent STAT3 and other signals, thus becoming a core mechanism for promoting chronic inflammation, fibrosis, and malignant transformation [97]. In chronic disease conditions such as fatty hepatitis and viral hepatitis, the trans-signaling axis driven by the continuous or excessive activation of ADAM17 becomes the core pathogenic factor [97]. Therefore, ADAM17 may serve as a critical regulator in balancing the dual protective and pathogenic roles of IL-6.

Beyond ADAM17, other members of this family also participate in the amplification of inflammatory effects. ADAM10 recruits and activates inflammatory cells by regulating the shedding of inflammatory mediators (CX3CL1, IL-6R). This prompts these cells to produce proinflammatory cytokines (such as TNF-α and IL-1β) and activate proinflammatory signaling pathways like NF-κB and MAPK, ultimately exacerbating the progression of acute liver injury and liver failure [100]. ADAM8 regulates the cleavage and release of membrane-bound factors such as TNF-α and CX3CL1 and activates focal adhesion kinase (FAK) and Src kinase (Src) signaling pathways. Consequently, it synergistically enhances the activation of classical inflammatory signaling pathways such as NF-κB and MAPK, ultimately promoting inflammatory responses and disease progression in acute liver injury and non-alcoholic steatohepatitis [101].

In addition to directly regulating inflammatory cytokines and chemokines locally in the liver, ADAM proteases can also indirectly influence the progression of liver disease by modulating immune homeostasis in the spleen. The liver and spleen are closely linked anatomically and functionally through the portal circulation, forming the “liver–spleen axis”. Activated immune cells (e.g., pro-inflammatory macrophages) and soluble factors originating from the spleen can migrate directly to the liver, thereby exacerbating hepatic inflammation, fibrosis, and even tumor progression [102]. Splenectomy has been shown to improve liver fibrosis and inhibit HCC growth, further confirming the critical role of the “liver–spleen axis” in liver pathophysiology [102]. Research indicates that ADAM10 is an important regulator of the splenic immune microenvironment, controlling the numbers of follicular helper T cells, the integrity of the follicular dendritic cell network, and the expression of the chemokine CCL21 in the spleen and draining lymph nodes [103]. Consequently, ADAM10 deficiency leads to a comprehensive immunodeficiency phenotype, including lymphoid structure disruption, impaired germinal center formation, and severely compromised antibody responses [103]. Furthermore, in models of acute systemic infection, ADAM17 cleaves CD122 on the surfaces of CD8+ T cells, resulting in more pronounced clonal expansion and a higher proportion of terminally differentiated effector cells among ADAM17-deficient CD8+ T cells in the spleen. This ultimately enhances pathogen clearance efficiency in the liver [104]. In summary, ADAM proteases serve as pivotal molecular nodes within the liver–spleen axis, profoundly influencing the initiation and progression of liver disease by coordinating local and systemic immune responses.

### 3.3. Regulation of HSCs and Progression of Hepatic Fibrosis

HSCs are the central effector cells in liver fibrosis. Upon hepatic injury, quiescent HSCs activate into myofibroblasts, which excessively synthesize extracellular matrix (ECM) components like collagen, leading to intrahepatic scarring [105]. Persistent injury drives disease progression to cirrhosis, characterized by irreversible structural alterations [105,106]. The ADAM protease family promotes fibrosis progression by modulating HSC activation-associated signaling pathways and ECM metabolism. Among these, ADAM10 and ADAM17 function as central regulators with multifaceted roles, while ADAM12, ADAM9, and ADAM8 contribute through distinct mechanisms (Figure 4) [68,86,107,108].

ADAM10 mainly exerts an anti-fibrotic role in liver fibrosis. ADAM10 negatively regulates the HGF, c-Met, and ERK signaling pathways by cleaving the c-Met receptor to inhibit the excessive activation of hepatic progenitor cells. Meanwhile, it prevents cholestatic hepatocyte necrosis by maintaining the expression of bile acid transporters (such as Mrp2, BSEP, and Oatp1b2). Ultimately, it inhibits the activation of hepatic stellate cells and collagen deposition, thereby effectively preventing the occurrence and development of liver fibrosis [90]. However, under certain conditions, ADAM10 can also exert a pro-fibrotic effect. In a metabolic dysfunction-associated steatohepatitis model, ADAM10 cleaves and releases membrane-bound bimodulated protein (pro-AREG), thereby activating the EGFR signaling pathway on hepatic stellate cells, and ultimately driving the activation of stellate cells and the occurrence of liver fibrosis. Its activity is directly negatively regulated by the reversion-inducing cysteine-rich protein with kazal motifs (RECK) protein. The overexpression of RECK in hepatocytes can specifically inhibit the enzymatic activity of ADAM10 [109]. Thus, the net effect of ADAM10 in fibrosis appears to be context-dependent, influenced by the disease etiology and the cellular microenvironment.

ADAM17 promotes HSC activation and liver fibrosis progression by cleaving and regulating multiple membrane proteins. Specifically, ADAM17 cleaves and releases the membrane-bound form of amphiregulin, thereby activating EGFRs and their downstream ERK and Akt signaling pathways, directly stimulating cell proliferation and survival of HSCs [110]. Furthermore, by cleaving the Notch1 receptor to initiate the Notch signaling pathway, the released intracellular domain is transported into the nucleus. This not only directly induces the transcription of fibrosis-related genes but also upregulates the expression of transforming growth factor-β receptor I (TGF-βI), significantly enhancing cellular responsiveness to TGF-β signaling and ultimately leading to excessive collagen deposition [111]. In NASH-related fibrogenesis, ADAM17-mediated ectodomain shedding of the Mer tyrosine kinase (MerTK) receptor on macrophages is reduced. This leads to accumulation of membrane-bound MerTK, which activates the ERK-TGFβ1 pathway, enhances TGFβ1 secretion, and consequently promotes HSC activation and collagen production [112].

During the progression of liver fibrosis, ADAM12 promotes fibrogenesis by integrating growth factor and cell adhesion signaling. Mechanistically, its extracellular domain binds directly to the TGF-β type II receptor, enhancing receptor internalization and converting TGF-β signaling into a sustained high-intensity response. This leads to persistent Smad2/3 activation and marked upregulation of profibrotic genes, including α-smooth muscle actin and types I/III collagen [113]. In parallel, the cytoplasmic tail of ADAM12 interacts with integrin-linked kinase (ILK). Upon extracellular matrix stimulation, such as by type I collagen, this complex is recruited to focal adhesions where it activates the PI3K/Akt survival pathway, significantly suppressing hepatic stellate cell apoptosis and maintaining their activated myofibroblast phenotype [114].

The role of ADAM8 in the progression of liver fibrosis is increasingly gaining attention. The study by Yang et al. clearly demonstrated that ADAM8, through its metalloproteinase activity, cleaves and activates various membrane-bound receptors and cytokines, thereby modulating the MAPK signaling pathway (including key components such as ERK, p38, and JNK). This process significantly promotes the activation, proliferation, and inflammatory response of hepatic stellate cells, ultimately driving the onset and progression of alcoholic liver fibrosis [108].

Most ADAM proteins demonstrate a definite role in promoting liver fibrosis. This effect is primarily mediated by directly targeting the activation and maintenance of HSCs. Specifically, ADAM proteases cleave and activate critical membrane proteins such as EGFR ligands, Notch receptors, and TGF-β-related components, thereby driving transformation of HSCs into myofibroblasts, promoting their proliferation, inhibiting their apoptosis, and enhancing their synthesis of extracellular matrix. Notably, certain members like ADAM10 exhibit anti-fibrotic effects under specific circumstances, indicating that the regulatory role of ADAM proteases in the fibrosis process is variable and depends on injury etiology, cellular context, and substrate selectivity. Beyond substrate specificity, potential anti-fibrotic mechanisms may include cleavage and release of protective soluble factors, negative feedback inhibition of pro-fibrotic signaling pathways, and regulation of cell differentiation toward a reparative phenotype [115].

### 3.4. Propelling Liver Cancer Pathogenesis

HCC represents the end-stage of malignant transformation in chronic liver disease. The ADAM protease family drives hepatocarcinogenesis and tumor progression by modulating tumor cell proliferation, migration, invasion, and interactions within the tumor microenvironment. Currently, ADAM8, ADAM9, ADAM10, ADAM12, ADAM15, ADAM17, and ADAM21 are known to play critical roles (Figure 5) [116].

In HCC, elevated ADAM8 expression correlated with poor patient prognosis. It promoted tumor cell proliferation, migration, invasion, and restrained apoptosis through upregulation of β1-integrin and activation of FAK and Src signaling pathways. Anti-ADAM8 interventions effectively retarded tumor progression [117,118,119].

ADAM9 is highly expressed in HCC tissues, where it promotes immune evasion by cleaving MHC class I polypeptide-related sequence A (MICA) to suppress NK cell activation and promotes ECM remodeling by degrading fibronectin. Its expression positively correlates with infiltration of regulatory T cells (Tregs) and cancer-associated fibroblasts (CAFs), enhancing the invasive and metastatic potential of HCC cells. Clinically, high ADAM9 expression is associated with poor prognosis and serves as both an independent prognostic risk factor and a potential therapeutic target [26]. Furthermore, ADAM9 is upregulated via the IL-6/JNK pathway, interacts with NADPH oxidase 1 (NOX1) to generate reactive oxygen species (ROS), and subsequently activates snail-driven epithelial–mesenchymal transition (EMT), thereby enhancing tumor cell invasiveness and metastatic potential [120,121].

ADAM10 has frequently been implicated as an oncogene in HCC, promoting tumor immune evasion through substrate shedding. Knockdown of ADAM10 suppresses proliferation, migration, and invasion of liver cancer cells. Clinical data analysis revealed that its elevated expression positively correlated with advanced tumor grade and increased risk of distant metastasis [122]. In HCC development, ADAM10 was frequently upregulated, and its expression level showed pronounced correlations with tumor grade, differentiation status, tumor size, and metastatic potential. These associations might be mediated through its proteolytic processing of basement membrane collagen type IV and CD44 [123]. In sorafenib resistance models, ADAM10 knockdown augments the antitumor efficacy of sorafenib by suppressing cell proliferation, migration, and invasion while promoting apoptosis. The underlying mechanisms potentially involve inhibition of PI3K/Akt phosphorylation, regulation of apoptosis-related proteins such as Caspase-8, Bcl-2, and Survivin, and modulation of extracellular matrix-degrading proteases, matrix metalloproteinase-2 (MMP-2) and MMP-9 [124].

ADAM12 accelerates HCC progression by activating Notch and TGF-β signaling pathways while upregulating Cyclin D1 expression, which drives G1/S phase transition and tumor cell proliferation. Moreover, it interacts with receptor for activated C kinase 1 (RACK1) to stimulate hepatic stellate cell-mediated stromal remodeling, enhancing invasive and metastatic capabilities. Clinically, elevated ADAM12 expression serves as an independent risk factor for reduced overall survival in liver cancer patients [125].

ADAM15 is aberrantly upregulated in HCC cells, showing a positive correlation with tumor grade. Its silencing suppresses proliferation and invasion of liver cancer cells, establishing this protease as a potential biomarker for unfavorable prognosis [122]. Mechanistically, it inhibited apoptosis through upregulation of the anti-apoptotic protein Bcl-2 and concurrent downregulation of the pro-apoptotic protein Bax. The protease also enhanced HCC cell proliferation, migration, and invasion by modifying EMT markers, exemplified by E-cadherin and Vimentin. Moreover, ADAM15 substantially affected tumor immune infiltration through altered recruitment of B cells, CD4+ T cells, and neutrophils, while controlling immune checkpoint genes including programmed death-ligand 1 (*PD-L1*) and cytotoxic T-lymphocyte-associated protein 4 (*CTLA-4*), thereby reshaping the tumor immune microenvironment [126].

ADAM17 mediates HCC progression through coordinated molecular actions. The protease cleaves EGFR ligands and initiates Notch signaling to enhance MMP-2 and MMP-9 production while modifying integrin β1 pathways to strengthen cellular adhesion and migration. Under hypoxic conditions, ADAM17 stimulates the EGFR/PI3K/Akt cascade, establishing sorafenib resistance. After radiotherapy, its increased expression in CD133-positive cancer stem cells amplifies radioresistance and metastatic potential. Therefore, pharmacological inhibition of ADAM17 offers a strategic approach to overcome treatment resistance and improve therapeutic outcomes [122,127].

ADAM21 plays a promoting role in HCC. In an in vivo mouse orthotopic transplantation model, ADAM21 knockdown effectively inhibited the growth of orthotopic liver tumors and reduced the tendency of intrahepatic metastasis [128]. In an in vitro cell function model, stable knockdown of ADAM21 by siRNA can significantly inhibit the proliferation, migration, and invasion abilities of highly metastatic KYN-2 hepatoma cells, and induce G0/G1 phase arrest and apoptosis of the cell cycle [128]. Clinical cohort analysis indicated that ADAM21 protein was highly expressed in liver cancer tissues and was significantly associated with adverse pathological features such as large tumor volume, poor tissue grade, and vascular invasion. It was an independent risk factor suggesting shortened overall survival and recurrence-free survival in multivariate analysis [128].

Overall, the ADAM family shapes an immunosuppressive microenvironment through regulating immune checkpoint molecule expression, recruiting monocyte-derived macrophages, and amplifying inflammatory responses. They also integrate EGFR and JAK/STAT signaling pathways, thereby promoting the malignant progression of HCC [122].

In HCC, the majority of studied ADAM proteases drive tumorigenesis and malignant progression. Their mechanisms of action fundamentally differ from those in fibrosis, primarily focusing on driving the autonomous malignant progression of tumor cells and remodeling the immunosuppressive microenvironment. ADAM proteins enhance tumor cell proliferation, invasion, and metastasis by promoting growth factor signaling, activating developmental pathways, inducing epithelial–mesenchymal transition, and regulating integrin-mediated adhesion and migration. Meanwhile, ADAM proteases contribute to tumor immune evasion by cleaving immunomodulatory molecules and modulating the expression of immune checkpoints, thereby establishing an immunosuppressive microenvironment that helps tumors evade immune surveillance. Furthermore, the ADAM family also participates in the formation of drug resistance by mediating the excessive activation of key survival pathways.

**Table 2:** Research Advances on ADAMs in Liver Pathogenesis.

ADAMs	Liver Disease	Function	Mechanisms	Types of Study	References
ADAM17	Cholestatic Liver Injury	Amplifying inflammatory responses and exacerbating hepatocyte necrosis with cholangiocyte proliferation	TNFR, IL-6R, and EGFR signaling pathways.	In vivo and clinical analysis	[89]
	Liver Fibrosis	Promotes HSC proliferation and liver fibrosis; negatively regulates TGF-β signaling and alleviates biliary injury	ERK/Akt, Notch1, TGF-β, and MerTK/ERK signaling pathways.	In vivo and in vitro	[110,111,112]
	Liver cancer	Enhances proliferative, migratory, and invasive capacities of HCC cells	The Notch signaling pathway, activation of MMP-2/9, VEGF secretion, and the EGFR/PI3K/Akt pathway.	In vivo and in vitro	[122,127]
ADAM10	Hepatocyte Injury and Regeneration	Maintains bile acid equilibrium and stimulates hepatocyte specialization	Inhibiting c-Met signaling to restrict excessive activation of liver progenitor cells and promoting the differentiation of liver progenitor cells into hepatocytes.	In vivo and in vitro	[90]
	Liver Fibrosis	Context-dependent; anti-fibrotic via c-Met, pro-fibrotic via EGFR	Regulation of bile acid transporters, negative regulation of the c-Met receptor, promotion of the AREG/EGFR signaling pathway	In vivo, in vitro, and clinical analysis	[90,109]
	Liver cancer	Cancer-promoting	Immune evasion, PI3K/Akt pathway, Notch pathway, EMT, MMP regulation.	In vivo, in vitro studies, and clinical analysis	[122,123,124]
ADAM8	Acute Liver Injury	Inhibits hepatocyte proliferation, angiogenesis, and hepatic metabolism	Inhibition of VEGF, down-regulation of CYP1A2, and inhibition of PCNA	In vivo	[91]
	Liver Inflammation	Amplifies Inflammatory Responses	TNF-α and NF-κB signaling pathways	In vivo and in vitro	[101]
	Liver Fibrosis	Promotes HSC activation and fibrosis	MAPK Signaling Pathway	In vivo and in vitro	[108]
	Liver cancer	Promotes tumor cell proliferation, migration, and invasion, and inhibits apoptosis	Inhibition of Bcl-2, Bax, Caspase-3, and p53, upregulation of PCNA, promotion of VEGF-A, and activation of the integrin-FAK-Src/Rho A axis	In vivo, in vitro studies, and clinical analysis	[117,118,119]
ADAM9	Acute Liver Injury	Hepatocyte proliferation in the CCl4-induced model; Promoting injury effect in the alcohol-induced model	IL-6/STAT3 Signaling Pathway	In vivo and in vitro	[92,93]
	Liver cancer	Enhances tumor cell invasion and metastasis; modulates tumor microenvironment	Induced via IL-6/JNK signaling, mediating the ROS/Snail axis, inhibiting NK cells, and promoting Treg infiltration.	In vivo, in vitro, and clinical analysis	[26,120,121]
ADAM12	Liver Fibrosis	Promotes HSC transdifferentiation and ECM remodeling	TGF-β signaling, integrin–PI3K–Akt pathway	In vitro	[113,114]
	Liver cancer	Promotes HCC proliferation and progression	The Notch/TGF-β signaling axis.	In vitro and clinical analysis	[125]
ADAM15	Liver cancer	Promotes HCC Cell Proliferation, Migration, and Invasion	Promotion of Bcl-2, N-cadherin, Vimentin, and Snail, suppression of Bax, E-cadherin, and ZO-1, and enhancement of immunosuppressive molecule expression.	In vitro and clinical analysis	[126]
ADAM21	Liver cancer	Suppresses HCC Tumor Growth and Intrahepatic Metastasis	Inhibit the movement, invasion and proliferation of HCC cells, and induce apoptosis (the specific pathways need to be further clarified)	In vivo, in vitro, and clinical analysis	[128]

## 4. Future Perspectives and Conclusions

The ADAM protease family, characterized by its zinc-dependent catalytic activity and multifunctional domains, plays a pivotal role in the pathogenesis of liver diseases. Through proteolytic “ectodomain shedding”, ADAM members are critically involved in the entire spectrum of hepatic pathology—from initial injury and inflammatory hepatitis to progressive fibrosis and ultimately HCC. Their regulation of inflammatory cascades, key signaling pathways, and the tumor microenvironment not only deepens our understanding of liver disease mechanisms but also unveils new avenues for early diagnosis, prognostic assessment, and targeted therapies.

### 4.1. The Potential of ADAM Proteases as Liver Disease-Specific Biomarkers

The distinct expression patterns and substrate specificities of ADAM family members hold significant promise as clinical biomarkers. In HCC, for instance, single-cell and spatial transcriptomic analyses have revealed aberrant activation of ADAM-associated signaling across multiple cell types within tumor tissues. Notably, endothelial cells and monocytes/macrophages are primary sources of ADAM signals, while malignant cells exhibit significantly stronger ADAM signaling than their benign counterparts. A molecular classifier developed through machine learning, based on ADAM signaling strength, serves as an independent prognostic predictor, capable of effectively stratifying high-risk patient subgroups [122].

However, a major translational challenge remains in deconvoluting the specific activity of individual ADAM members due to substantial substrate overlap and functional redundancy. A more promising biomarker strategy may involve quantifying specific shedded fragments, such as soluble CD23 (sCD23) for ADAM10 and soluble CD163 (sCD163) for ADAM17, as these cleavage products directly reflect functional protease activity in vivo and may correlate more closely with disease state than total protein levels. For example, plasma levels of ADAM17-shed sCD163 are significantly elevated in patients with non-alcoholic steatohepatitis (NASH) and show a strong negative correlation with hepatic CD163 protein expression, presenting a potential non-invasive biomarker for distinguishing NASH from simple steatosis and for staging fibrosis [129].

Nevertheless, several core challenges must be addressed to realize the diagnostic potential of ADAM proteases. (1) Tissue Specificity: As widely expressed proteases, elevated ADAM activity (e.g., ADAM17) is observed in various non-hepatic diseases such as rheumatoid arthritis [130] and sepsis [131]. To attribute serum signals specifically to hepatic pathology, future strategies could employ multi-marker panels combining ADAM-shed substrates with classic liver injury markers. Alternatively, immuno-capture methods to enrich hepatocyte-derived exosomes [132] and subsequent detection of ADAM cargo or activity within these vesicles could provide a more liver-specific readout. (2) Activity vs. Abundance: The pathological significance of ADAM depends on its catalytic activity, not merely its abundance [133]. Traditional immunoassays often fail to distinguish between inactive zymogens, active enzymes, and inhibitor-complexed forms. Techniques such as substrate probes based on Förster Resonance Energy Transfer and active site-directed capture provide new pathways for direct detection of enzyme activity [134]. (3) Spatiotemporal Dynamics: The cellular sources and functional roles of ADAM proteases may shift across different stages and etiologies of liver disease. Integrating techniques such as single-cell sequencing, spatial transcriptomics/proteomics, and longitudinal serum cytokine/sheddome analysis will be essential to map these dynamics and identify the most context-relevant ADAM member or substrate for diagnostic use.

### 4.2. Therapeutic Strategies Targeting ADAM Proteases in Liver Diseases

Therapeutic interventions targeting specific ADAM proteases have shown considerable efficacy in preclinical models, establishing a foundation for clinical translation. Representative agents include the ADAM9 inhibitor CCL347 and the ADAM17 inhibitor ZLDI-8. CCL347 inhibits ADAM9 enzymatic activity, reducing the production of soluble MICA (sMICA). This alleviates sMICA-mediated inhibition of NK cells and activates tumor immunity to effectively eliminate HCC cells, while demonstrating minimal cytotoxicity [135]. ZLDI-8, a selective ADAM17 inhibitor, exerts its therapeutic effects through multiple mechanisms. It suppresses Notch1 cleavage and downstream NICD-mediated transcription of anti-apoptotic and EMT-related genes; attenuates integrin β1/β3 and ILK signaling [136,137]; reverses TGF-β1-induced EMT; and sensitizes HCC to sorafenib treatment [138].

Despite these promising preclinical results, significant hurdles remain for clinical translation. (1) Functional Redundancy and Compensatory Mechanisms: A major challenge is the inherent redundancy within the ADAM family [9]. Inhibiting one member (e.g., ADAM17) may lead to compensatory upregulation or activation of another (e.g., ADAM10 or ADAM9), potentially limiting therapeutic efficacy and driving adaptive resistance. This redundancy operates at the level of shared substrates, convergent downstream pathways (e.g., EGFR activation), and cell-type-specific substitution [139]. Future drug development must therefore prioritize the design of highly selective inhibitors and strategically explore rational combination therapies—either targeting multiple critical ADAMs simultaneously or co-inhibiting an ADAM protease and its key downstream pathway. (2) Precision Targeting: A more fundamental strategy involves identifying and targeting non-redundant, context-dependent nodes—specific substrate–enzyme pairs or proteolytic events that are exclusively governed by a particular ADAM member in a given disease setting (e.g., specific cleavage events in activated HSCs or HCC stem cells). This requires a deeper mechanistic understanding of the precise roles of individual ADAMs in specific liver cell types and disease stages. (3) Beyond catalytic inhibition: Most current strategies focus on inhibiting the metalloproteinase activity. However, the non-proteolytic functions of ADAMs, such as those mediated by their disintegrin and cytoplasmic domains in cell adhesion and intracellular signaling, represent underexplored therapeutic avenues. Modulating these functions could offer novel therapeutic opportunities with different safety profiles.

### 4.3. Future Research Directions and Challenges

To advance the field, future research should focus on the following frontiers: (1) Employing cell-type-specific conditional knockout mouse models (e.g., in hepatocytes, HSCs, Kupffer cells, or liver sinusoidal endothelial cells) is essential to dissect the autonomous versus non-autonomous roles of specific ADAM members in liver pathophysiology. This will clarify whether therapeutic targeting should be directed at parenchymal or non-parenchymal cells. (2) ADAM proteases do not operate in isolation. They are embedded in complex regulatory networks involving other protease systems (e.g., MMPs, sheddases), endogenous inhibitors (TIMPs), and signaling feedback loops. Utilizing systems biology approaches and multi-omics integration will be key to understanding this network and predicting the consequences of its perturbation. (3) Bridging the gap between mechanistic discovery and clinical application requires robust validation. Prospective clinical studies are needed to correlate ADAM or substrate levels/activity with disease progression, treatment response, and outcome. Furthermore, the development of clinically applicable activity-based probes or imaging agents for ADAMs could revolutionize patient stratification and treatment monitoring. (4) Beyond small-molecule inhibitors, emerging therapeutic modalities such as monoclonal antibodies, proteolysis-targeting chimeras (PROTACs) for ADAM degradation, or gene-silencing approaches (siRNA, antisense oligonucleotides) may offer improved specificity and novel mechanisms to target this protein family.

### 4.4. Conclusions

In conclusion, this review systematically synthesizes the sophisticated domain architecture, regulated activation, and diverse substrate repertoire of the ADAM protease family, highlighting their multifaceted and context-dependent roles across the spectrum of liver diseases. Their roles span the regulation of hepatocyte fate and injury, inflammation, fibrogenesis, and oncogenesis, highlighting their position as critical hubs in liver pathophysiology. The dual, sometimes contradictory, functions of individual ADAMs (e.g., ADAM10 in fibrosis) emphasize the complexity of this family and the necessity for precise, context-aware therapeutic strategies. Future progress hinges on moving from a focus on individual ADAMs to understanding their integrated network, leveraging advanced technologies for biomarker and inhibitor development, and fostering translational research that directly connects mechanistic insights to patient care. We anticipate that this summary will provide valuable guidance for future research, facilitating the development of ADAM-based diagnostic biomarkers, targeted therapeutics, and clinical translation strategies for the benefit of patients with liver diseases.

## Data Availability

No new data were created or analyzed in this study. Data sharing is not applicable to this article.

## Abbreviations

The following abbreviations are used in this manuscript:

Aβ	Amyloid-β
ABCA1	ATP-binding cassette transporter A1
ACE2	Angiotensin-Converting Enzyme 2
ADAM	A Disintegrin and Metalloproteinase
APAP	Acetaminophen
APP	Amyloid Precursor Protein
AREG	Amphiregulin
Bax	Bcl-2-associated X protein
Bcl-xL	B-cell lymphoma-extra large
Bcl-2	B-cell lymphoma 2
BDL	Bile duct ligation
Bim	Bcl-2-like protein 11
BSEP	Bile Salt Export Pump
CAFs	Cancer-Associated Fibroblasts
CTLA-4	Cytotoxic T-Lymphocyte-Associated Protein 4
CYP1A2	Cytochrome P450 1A2
CYP2E1	Cytochrome P450 2E1
DAMPs	Damage-Associated Molecular Patterns
Dll1	Delta-like 1
ECM	Extracellular Matrix
EGF	Epidermal Growth Factor
EGFR	EGF Receptor
EMT	Epithelial–Mesenchymal Transition
EphA3	Ephrin receptor A3
ErbB2	Erythroblastic leukemia viral oncogene homolog 2
ERM	Ezrin, radixin, moesin
FAK	Focal Adhesion Kinase
gp130	glycoprotein 130
HA	Hyaluronic Acid
HB-EGF	Heparin-binding EGF-like Growth Factor
HCC	Hepatocellular Carcinoma
Hes5	Hairy and enhancer of split 5
HGF	Hepatocyte Growth Factor
HSCs	Hepatic Stellate Cells
IGFBP-3	Insulin-like Growth Factor-Binding Protein-3
IGF-1	Insulin-like Growth Factor-1
IGF-1R	Insulin-like growth factor-1 receptor
ILK	Integrin-Linked Kinase
LDLR	Low-Density Lipoprotein Receptor
Mcl-1	Myeloid Cell Leukemia 1
MerTK	Mer Tyrosine Kinase
MICA	MHC Class I Polypeptide-Related Sequence A
mIL-6R	Membrane-bound IL-6R
MMP-2	Matrix Metalloproteinase-2
Mrp2	Multidrug Resistance-associated Protein 2
NICD	Notch Intracellular Domain
NOX1	NADPH Oxidase 1
NF2	Neurofibromin 2
PAMPs	Pathogen-Associated Molecular Patterns
PBC	Primary biliary cholangitis
PCNA	Proliferating Cell Nuclear Antigen
PCSK9	Proprotein Convertase Subtilisin/Kexin Type 9
PD-L1	Programmed Death-Ligand 1
PKC	Protein Kinase C
PKC-α	Protein Kinase C alpha
PMA	Phorbol 12-myristate 13-acetate
PPP1R14D	Protein Phosphatase 1 Regulatory Subunit 14D
PrPC	Cellular Prion Protein
PSC	Primary sclerosing cholangitis
pSTAT3	Phosphorylated Signal Transducer and Activator of Transcription 3
RACK1	Receptor for Activated C Kinase 1
RAS	Rat Sarcoma virus oncogene homolog
RECK	Reversion-inducing Cysteine-rich protein with Kazal motifs
ROS	Reactive Oxygen Species
sACE2	Soluble ACE2
sIL-6R	Soluble IL-6R
sMICA	Soluble MICA
sPrPC	Soluble PrPC
Src	Src kinase
sTNF-α	Soluble TNF-α
sTNFR1	Soluble TNF Receptor 1
sVCAM-1	Soluble VCAM-1
TACE	TNF-α Converting Enzyme
TGF-α	Transforming Growth Factor-alpha
TGF-βI	Transforming growth factor-β receptor I
TNFR1	TNF Receptor 1
Tregs	Regulatory T cells
VCAM-1	Vascular Cell Adhesion Molecule-1
VEGF	Vascular Endothelial Growth Factor
